# Association of Obesity With Type 2 Diabetes Mellitus: A Hospital-Based Unmatched Case-Control Study

**DOI:** 10.7759/cureus.52728

**Published:** 2024-01-22

**Authors:** Sakhawat Ali, Rizwana Hussain, Rohaib A Malik, Raheema Amin, Muhammad N Tariq

**Affiliations:** 1 Medicine, Ayub Medical College, Abbottabad, PAK; 2 Community Medicine, Ayub Medical College, Abbottabad, PAK

**Keywords:** case-control study, body mass index, obesity, type 2 diabetes mellitus, diabetes mellitus

## Abstract

Background

The prevalence of type 2 diabetes mellitus (T2DM) and obesity is alarmingly increasing with the accessibility of the modern lifestyle. This study aimed to assess the association of obesity with T2DM among the patients visiting the Medicine Department of Ayub Teaching Hospital, Abbottabad, Pakistan.

Method

This hospital-based, unmatched case-control study was conducted from March 2022 to September 2022. A total of 200 patients (age ≥ 18) (100 cases and 100 controls) were recruited. Those patients with a history of T2DM were selected as cases, and those without diabetes were selected as controls after taking informed written consent. Patients with BMI ≥ 25 were considered obese. Data were collected through a non-probability convenience sampling technique using a self-structured non-validated questionnaire. Data were organized and analyzed through IBM SPSS Statistics for Windows, version 26.0 (IBM Corp., Armonk, NY).

Results

We found a significant positive association of obesity with T2DM with a crude odds ratio of 3.6 (95% CI: 2.0-6.6), a p-value of 0.000, and an adjusted odd ratio of 3.7 (95% CI: 1.9 - 7.1), with a p-value of 0.004 (adjusted for potential confounders, including gender, age group, stress, and status of physical activeness) using a logistic regression model.

Conclusion

It is concluded that obesity is strongly associated with developing T2DM and lack of physical activity, people over 45 years, and males with obesity have a higher chance of developing T2DM.

## Introduction

Type 2 diabetes mellitus (T2DM) is a major “modern preventable pandemic” with a multifactorial impact on public health and the global economy [1]. It is alarmingly increasing with the convenience of modern life, lack of physical activity, poor diet, and most importantly, the increasing prevalence of obesity. Worldwide, its prevalence in the adult population, as estimated by the International Diabetes Federation (IDF) in 2021, is about 537 million (10.5%) compared to 108 million in 1980. It is estimated that diabetes contributed to 6.7 million (12.2%) deaths globally in 2021, according to the IDF, as compared to 1.5 million deaths in 2019, according to the WHO [2,3]. In Pakistan, diabetes cases are significantly increasing, as the national prevalence estimated by IDF in 2021 is 33 million (30.8%), which is a 70% increase since 2019 [2,4]. Globally, Pakistan ranked the 1st country with the most prevalence of diabetes mellitus and 4th among the countries with the most significant number of the adult diabetic population [2,4]. Although no current data are available in the Khyber Pakhtunkhwa (KPK) province, according to a study in 2016, 9.2% of males and 11.60% of females have diabetes [5].

Obesity is the well-established modifiable risk factor for T2DM, and unfortunately, it is also a modern pandemic with about 1.9 billion of the entire population of the world being overweight; among them, more than 0.6 billion are obese [6]. In Pakistan, about 20-30% of the adult population are overweight, and about 5-9% of them are obese with the standard cutoff of BMI ≥ 30 kg/m^2^ [7-9]. KPK has the highest prevalence among other provinces with 41% overweight and 14% obese adult population [8].

T2DM is caused by chronic decreasing tissue insulin sensitivity, known as insulin resistance, combined with reduced synthesis of insulin due to the loss of insulin-producing pancreatic beta cells resulting in hyperglycemia. In most cases (about 60-90%) of insulin-resistant types of disease, obesity invariably presents [10,11]. Due to this strong relationship, diabesity has been coined to represent their interdependence [12]. As body weight or intra-abdominal fat increases, body tissues gradually develop insulin resistance even without the disease. Earlier in the disease, patients present with increased thirst, urination, and delayed wound healing. Prolonged periods of hyperglycemia cause damage to various organs, especially microvascular, resulting in retinopathy, peripheral neuropathy, and nephropathy. Macrovascular involvement increases the risk of cardiovascular diseases and cerebrovascular accidents [13].

Earlier studies [14,15] suggest that weight loss and lifestyle modifications improve insulin sensitivity and mitigate the disease progression. However, the real problem in its prevention is the late presentation with complications. It looks crucial to evaluate the association with different comorbid conditions, especially obesity, to identify the individuals at high risk in the local populations. Until now, few or no such study is conducted in the KPK province, especially in the city of Abbottabad, to know the association between the two comorbid conditions in the local population. Our study aimed to assess the association of T2DM with its most substantial risk factor, i.e., obesity, through a case-control study method, in patients visiting the Medicine Department of Ayub Teaching Hospital, Abbottabad.

## Materials and methods

Study design, setting, and sampling

A hospital-based, un-matched case-control study method was designed and conducted in the largest tertiary care hospital of the Hazara Division, Ayub Teaching Hospital (ATH), Abbottabad. Patients with a history of T2DM and/or on anti-diabetic medication were selected as cases, and an equal number of patients without diabetes were selected as controls (1:1). Two hundred eligible and willing patients, 100 cases and 100 controls, were recruited in this study. Data (primary) were collected through a non-probability convenience sampling technique, using a self-structured non-validated questionnaire after obtaining informed written consent from the participants. Body mass index (BMI) was used as the index of obesity. Patients with BMI < 18.5 kg/m2 were considered underweight, between 18.5 and 22.9 kg/m2 as normal weight, between 23 and 24.9 kg/m2 as overweight, ≥25 to 29.9 kg/m2 as obese I, and ≥30 kg/m2 were considered obese II (WHO’s Asian cutoff value). Male and female participants aged ≥ 18 years and without any weight-losing surgery were included. Participants with a history of type 1 diabetes mellitus, under 18 years, and pregnant females were excluded. Participants with any kind of physical activity ≥ 30 minutes at least three times a week were considered physically active, otherwise physically inactive. The study was conducted from March 2022 to September 2022.

Data collection

Data were collected from the participants through face-to-face interviews and from their past medical reports by the investigators themselves. Essential demographic data (like gender, age, marital status, level of education, and occupation), lifestyle (physical activeness, body weight maintenance, daily work hours, deity preference, and daily meals), and anthropometric data (weight, height, and BMI) were obtained by self-structured questionnaire. Participants were selected as case or control after asking about a history of diabetes and/or on medication. Exposure to obesity was investigated by asking patients about their weight and height, history of weight gained or lost before, and also investigating their past medical reports. Weight (nearest to 1 kg) and height (nearest to 1 cm) were also measured during the data collection by a self-made stadiometer and weight scale.

Data analysis

Data were entered, organized, and analyzed in IBM SPSS Statistics for Windows, version 26.0 (IBM Corp., Armonk, NY). The continuous variables, like age, were reported in means plus standard deviation (SD), and the categorical variables were represented in frequencies with percentages. Essential demographic characteristics, lifestyle behaviors, and anthropometric data of cases and controls were compared using independent samples t-test and chi-square (X2) test for continuous and categorical variables, respectively. The association of obesity with T2DM was assessed first by estimating the crude odds ratio (OR) with 95% CI. Then we stratified data into gender (male and female), age group (up to 45 years and above 45 years), and status of physical activeness (physically active and lack of physical activity) and estimated odd ratios. Secondly, we estimated the adjusted odd ratio using a binary logistic regression model that adjusted the odd ratio for the possible confounders - gender, age group, and status of physical activeness. We also estimated the odd ratios for different categories of BMI by using a multivariate logistic regression model, which was also adjusted for gender, age group, and physical activity status. All the analytical tests were two-tailed, and all p-values less than 0.05 were accepted as significant.

## Results

Data from all 200 participants were included in the final analysis; among them, 98 (49%) were male and 102 (51%) were female. A total of 74% of the cases and 44% of controls were obese with BMI ≥ 25 kg/m2. Among the obese, 62.7% (74 out of 118 patients) had diabetes. The basic demographic characteristics, lifestyle behaviors, and anthropometric data of cases and controls, as shown in Table 1, were compared, and it was found that cases were more likely higher in age (mean ± SD age: 53.25 ± 11.8 in the case group vs. 41.46 ± 14.8 in the control group; p-value = 0.000) and had higher body mass index (mean ± SD BMI: 27.44 ± 4.3 in the case group vs. 24.19 ± 4.3 in the control group; p-value = 0.000). Patients in the case group were also mostly illiterate, aged above 45 years, had a BMI above 25 kg/m2, and had a lack of physical activity compared with those in the control group, with all p-values < 0.003.

**Table 1:** Essential demographic characteristics, lifestyle behaviors, and anthropometric measures of cases and control groups. P-values are calculated using t-tests for quantitative variables and the chi-square test (X2) for categorical variables.

Variables		Cases (with diabetes), N (%) = 100 (100%)	Controls (without diabetes), N (%) = 100 (100%)	P-value
Gender, N (%)	Male	46 (46%)	52 (52%)	0.396
	Female	54 (54%)	48 (48%)	
Age (means ± SD)		53.25 ± 11.8	41.46 ± 14.8	0.000
Age group, N (%)	Up to 45 years	29 (29%)	62 (62%)	0.000
	Above 45 years	71 (71%)	38 (38%)	
Educational status, N (%)	Illiterate	54 (54%)	35 (35%)	0.000
	Up to 10^th^ grade	35 (35%)	37 (37%)	
	Above 10^th^ grade	11 (11%)	28 (28%)	
BMI (means ± SD kg/m^2^)		27.44 ± 4.3	24.19 ± 4.3	0.000
Obesity (BMI ≥ 25 kg/m^2^)	Obese (≥ 25)	74 (74%)	44 (44%)	0.000
	Non-obese (< 25)	26 (26%)	56 (56%)	
BMI category, N (%)	<18.5 kg/m^2^	2 (2%)	10 (10%)	0.000
	18.5-22.9 kg/m^2^	13 (13%)	28 (28%)	
	23-24.9 kg/m^2^	11 (11%)	19 (19%)	
	25-29.9 kg/m^2^	59 (59%)	39 (39%)	
	30 kg/m^2^ & above	15 (15%)	4 (4%)	
Status of physical activity, N (%)	Physically active	29 (29%)	52 (52%)	0.001
	Lack of physical activity	71 (71%)	48 (48%)	
Stress in life, N (%)	Yes	46 (46%)	42 (42%)	0.526
	No	53 (53%)	58 (58%)	

On crude analysis, as displayed in Table 2, we found a significant positive association with a crude odds ratio (COR) of 3.6 (95% CI: 2.0-6.6), with a p-value of 0.000. Further, we stratified data in genders (male and female), age groups (up to 45 years and above 45 years), and status of physical activeness (physically active and lack of physical activity) to observe their potentially confounding effects and estimated the COR, i.e., 5.5 (95% CI: 2.3-13.3) for males, 2.6 (95% CI: 1.1-5.9) for females, 3.6 (95% CI: 1.3-9.6) for ages up to 45 years, 4.5 (95% CI: 2.0-10.7) for age above 45 years, and 5.2 (95% CI: 2.3-11.7) for lack of physical activity group, with all p-values < 0.021.

**Table 2:** Crude analysis, stratifying, and adjusted analysis using a multivariate logistic regression model to assess the association of obesity with type 2 diabetes mellitus. ^a^: Adjusted for gender, age group, and status of physical activeness by using a multivariate logistic regression model.

Variables		Analysis with crude odds ratio (COR)			Analysis with adjusted odds ratio (AOR)^a^	
		COR (with 95% CI)		P-value	AOR (with 95% CI)	P-value
Obesity (≥25 kg/m^2^)		3.6 (2.0-6.6)		0.000	3.7 (1.9-7.1)	0.000
On stratification						
Gender	Male	5.5 (2.3-13.3)	0.000		1.2 (0.6-2.3)	0.547
	Female	2.6 (1.1-5.9)	0.021		Reference	
Age group	Up to 45 years	3.6 (1.3-9.6)	0.009		Reference	0.000
	Above 45 years	4.6 (2.0-10.7)	0.000		3.9 (2.1-7.6)	
Status of physical activity	Physically active	2.1 (0.8-5.3)	0.113		Reference	0.033
	Physically inactive	5.2 (2.3-11.7)	0.000		2.0 (1.1-3.9)	

As displayed in Table 2, we also performed an adjusted analysis by using a multivariate logistic regression model that adjusted for gender, age group, and status of physical activeness and found an adjusted odd ratio of 3.7 (95% CI: 1.9-7.1), with a p-value of 0.000.

Furthermore, we also assessed the association with different BMI categories with normal weight category (BMI: 18.5-22.9 kg/m2) as a reference variable using a multivariate logistic regression model and found that the association is even stronger over higher BMI categories, with the adjusted odds ratio of 3.2 (95% CI: 1.4-7.4) in obese I category (BMI: 25-29.9 kg/m2), with a p-value of 0.007, and that of 8.8 (95% CI: 2.2-34.7) in obese II category (BMI: 30 kg/m2 or above), with a p-value of 0.000, as shown in Table 3.

**Table 3:** The association of different BMI categories, gender, age group, and physical activity status with type 2 diabetes mellitus. ^a^: Adjusted for gender, age group, and status of physical activeness by using a multivariate logistic regression model.

Variables		Analysis with crude odds ratio (COR)		Analysis with adjusted odds ratio (AOR)^a^	
		COR (with 95% CI)	P-value	AOR (with 95% CI)	P-value
BMI category (kg/m^2^)	Normal weight (18.5-22.9)	Reference			
	Underweight (<18.5)	0.4 (0.1-2.3)	0.318	0.7 (0.1-3.8)	0.641
	Overweight (23-24.9)	1.2 (0.5-3.4)	0.663	1.0 (0.3-2.8)	0.964
	Obese I (25-29.9)	3.3 (1.5-7.1)	0.003	3.2 (1.4-7.4)	0.007
	Obese II (30 or above)	8.1 (2.2-29.2)	0.001	8.8 (2.2-34.7)	0.002
Gender	Male	0.8 (0.4-1.4)	0.396	0.8 (0.4-1.5)	0.457
	Female	Reference			
Age group	Up to 45 years	Reference			
	Above 45 years	3.9 (2.2-7.2	0.000	4.0 (2.1-7.9)	0.000
Status of physical activeness	Active	Reference			
	Inactive	2.6 (1.5-4.8)	0.001	1.98 (1.03-3.82)	0.041

## Discussion

Our study found a positive statistically significant association of obesity with T2DM with an adjusted odds ratio of 3.7 after adjusting for gender, age group, and status of physical activity. We also observed statistically significant increasing odds ratios for T2DM with increasing levels of BMI category, with an adjusted odds ratio of 3.2 in the obese I category and 8.1 in the obese II category.

These results are consistent with past studies conducted in other parts of the world that assessed the association of BMI with T2DM. For instance, in a large case-control study conducted on the US population using data from an electronic health records system, a statistically significant association of body mass index with T2DM was found, with adjusted odds ratios of 1.6 (95% CI: 1.5-1.8) in overweight, 3.2 (95% CI: 2.9-3.5) in obesity I category, and 5.8 (95% CI: 5.3-6.5) in obesity II category, increasing odds ratios with higher levels of BMI [16]. There is a slightly different result in our study and the odds ratio of 1.3 in the overweight category is statistically not significant and should be interpreted with our limited sample compared with the large sample (37,356) in the US population study, and may also be due to the WHO Asian cutoff BMI ≥ 25 in our study.

A meta-analysis conducted at the School of Public Health and Preventive Medicine, Monash University, Australia, using 18 cohort studies from different regions (eight from the USA, five from Europe, and five from Asia-Pacific regions) estimated the relative risk for the development of type 2 diabetes in overweight and obese adults in various populations with a total sample of 590,251 adult individuals. Most of the studies included in that meta-analysis used a BMI cut-off point of 23 kg/m2, and few used the cut-off point of 25 kg/m2 for overweight and obesity. The study also found a positive association with the overall adjusted RRs of 7.28 for T2DM with obesity and 2.99 for overweight as compared to individuals with normal body weight (BMI = 18-24.9 kg/m2) [17]. Similarly, a sub-analysis of the US national survey (1999-2006) found 80.3% of type 2 diabetic patients were overweight or higher with BMI ≥ 25 and also noted that the prevalence of diabetes increased with the increasing body weight as 8% of the diabetics were found with normal body weight (BMI < 25), 15% with overweight, 23% with class 1 obesity, 33% with class 2 obesity, and 43% with class 3 obesity (BMI ≥ 40) [18]. A population-based cohort study in China also found that there was a 23% increased risk of T2DM development with each kg/m2 increase in BMI and also noted a linear relationship of obesity (BMI) with the risk of T2DM development in all age groups, which was stronger in younger age [19].

Although our study results are consistent with those in other parts of the world, it does not support the sub-analysis of the second National Diabetic Survey of Pakistan, conducted in 2017. It was noted in the sub-analysis that there was a higher rate of T2DM in the normal BMI group and concluded that the association of BMI ≥ 25 with T2DM was much less than in other parts of the world [20].

It is necessary to note that our study has a few limitations. Although we adjusted the odds ratio for gender, age group, and physical activity status, we did not include the family history of T2DM in our study. Also, we adjusted the OR but did not match the cases and controls over demographic and other characteristics. Furthermore, it is crucial to highlight that our study findings should be interpreted in light of the relatively small sample size, which may impact their generalizability.

## Conclusions

Our study concluded that obesity is strongly associated with developing T2DM, especially in higher body mass index categories. The association remained highly significant after adjustment for gender, age group, and status of physical activity. However, to declare obesity is a cause of T2DM, a temporality issue along with other criteria needs investigation. Furthermore, lack of physical activity and age over 45 years are also independently associated with T2DM. These findings highlight obesity, lack of physical activity, and age over 45 years as potential risk factors for T2DM. It also emphasizes the need for interventions targeted to prevent and manage obesity to reduce the risk of T2DM. Also, it highlights the importance of physical activity in mitigating the disease risk.
